# Is it time to use real-world data from primary care in Alzheimer’s disease?

**DOI:** 10.1186/s13195-020-00625-2

**Published:** 2020-05-18

**Authors:** Anna Ponjoan, Josep Garre-Olmo, Jordi Blanch, Ester Fages, Lia Alves-Cabratosa, Ruth Martí-Lluch, Marc Comas-Cufí, Dídac Parramon, María Garcia-Gil, Rafel Ramos

**Affiliations:** 1 Vascular Health Research Group (ISV-Girona), Jordi Gol Institute for Primary Care Research (IDIAPJGol), Barcelona, Catalonia Spain; 2 Girona Biomedical Research Institute (IDIBGi), Girona, Catalonia Spain; 3 Autonomous University of Barcelona, Bellaterra (Cerdanyola del Vallès), Catalonia Spain; 4 Primary Care Services, Catalan Institute of Health (ICS), Girona, Catalonia Spain; 5 Department of Medical Sciences, School of Medicine, Campus Salut, University of Girona, Girona, Catalonia Spain; 6 IDIAPJGol, c/ Maluquer Salvador, 11 baixos, 17002 Girona, Catalonia Spain

**Keywords:** Family physician, Electronic medical records, Epidemiology, General practitioner, Dementia

## Abstract

**Background:**

The analysis of real-world data in clinical research is rising, but its use to study dementia subtypes has been hardly addressed. We hypothesized that real-world data might be a powerful tool to update AD epidemiology at a lower cost than face-to-face studies, to estimate the prevalence and incidence rates of AD in Catalonia (Southern Europe), and to assess the adequacy of real-world data routinely collected in primary care settings for epidemiological research on AD.

**Methods:**

We obtained data from the System for the Development of Research in Primary Care (SIDIAP) database, which contains anonymized information of > 80% of the Catalan population. We estimated crude and standardized incidence rates and prevalences (95% confidence intervals (CI)) of AD in people aged at least 65 years living in Catalonia in 2016.

**Results:**

Age- and sex-standardized prevalence and incidence rate of AD were 3.1% (95%CI 2.7–3.6) and 4.2 per 1000 person-years (95%CI 3.8–4.6), respectively. Prevalence and incidence were higher in women and in the oldest people.

**Conclusions:**

Our incidence and prevalence estimations were slightly lower than the recent face-to-face studies conducted in Spain and higher than other analyses of electronic health data from other European populations. Real-world data routinely collected in primary care settings could be a powerful tool to study the epidemiology of AD.

## Background

Alzheimer’s disease (AD) is the most common form of dementia, a major worldwide public health concern, and a huge burden to patients, families, and public health systems. The lack of any effective treatment to tackle it and the aging of the population—age is the main risk factor for AD—may partly explain the forecasted strong increase in the worldwide prevalence of AD expected for the next decades [1]. However, some recent studies have shown a decline in the incidence or prevalence of AD in high-income countries, and improved education levels and better management of cardiovascular risk factors have been suggested as driving factors for such declining pattern [2, 3]. Therefore, updated regional or national epidemiology of AD is urged to adapt public policies to the secular and geographical trends of the disease [4]. Nevertheless, updated estimates of AD prevalence and incidence in Europe are scarce [5]; for example, epidemiological studies in Spain are based on data collected in face-to-face surveys conducted in the 1990s or 2000s [6–10]. Real-world data refers to observational data routinely collected in healthcare settings derived from multiple sources: electronic health records (EHR), disease registries, claims and billing data, and even data gathered through personal devices and health applications. Real-world data collected in primary care settings might provide a new opportunity to study the epidemiology of AD; general practitioners, as gatekeepers of the healthcare services, might provide a large number of cases compared to other clinical databases. However, epidemiological studies based on real-world data from primary care have hardly addressed specific dementia subtypes, such as AD [11–13]. This lack of evidence may be related to the high level of under-diagnosis of dementia or AD cases in primary care [14]. However, recent reports stated good accuracy of diagnoses of dementia and AD recorded in primary care databases for research purposes [15–17]. Therefore, we hypothesized that real-world data could provide regular updates on AD prevalence and incidence estimates, at reduced economical costs.

We sought to estimate the crude and standardized prevalence and incidence rates of AD in Catalonia using data from a large primary care database, including stratified results by age and sex. Additionally, we aimed to examine the suitability of real-world data to study the epidemiology of AD, comparing our estimates with those from previous face-to-face studies.

## Methods

This is a population-based study conducted in Catalonia using a large EHR database from primary care.

### Settings

The Catalan public health system provides universal health coverage to all citizens and attends patients within both primary and secondary care. General practitioners and primary care nurses, as the gatekeepers of the healthcare services, assess cognition, functional ability, and independence of patients with memory complaints reported by themselves and their relatives or detected by the general practitioner. Cognitive assessment is also performed in patients without memory complaints when they are institutionalized due to chronic complex morbidity, or included in specific healthcare programs (for example, the “at-home care program”). Assessment mainly consists in the administration of one cognitive test (such as Mini-Mental Status Examination (MMSE) [18] or Pfeiffer test [19]), one functional ability and independence tests (Barthel Index [20], Lawton Scale [21], Katz Index [22], or Blessed Dementia Rating Scale [23]), and, in some cases, one depression scale. Diagnosis of AD can be made by general practitioners, but about 80–90% of the dementia and AD diagnoses recorded in SIDIAP were made by a specialist [16, 17]. Prescription of anti-dementia drugs can be requested by the general practitioner but requires an external approval from a geriatrician, psychiatrist, or neurologist; before 2014, the approval came from the advisory board that evaluated all patients with dementia and prescribed pharmacological treatment. Therefore, all patients prescribed with anti-dementia drugs have been evaluated by one or several experts. The renewal of anti-dementia drug prescription is carried out in the primary care services.

### Data sources

We obtained data from the Information System for Research in Primary Care (SIDIAP database) [24], which gathers information from the primary care services in a structured way so it can be reliably used for research. SIDIAP contains data from about 6 million patients (> 80% of the Catalan population and 15% of the Spanish population) attended by 1365 general practitioners in the primary care settings of the Catalan Health Service (Institut Català de la Salut). SIDIAP contains anonymized longitudinal medical records related to demographics, symptoms, diagnoses (coded according to the *International Classification of Diseases, 10th revision* (ICD-10)), and laboratory tests. SIDIAP is linked with other external databases (and anonymization is preserved); we were able to use information from the register of mortality and the pharmacy-invoicing database provided by the Catalan Health Service, in which medications are recorded using the *Anatomical Therapeutic Chemical* (ATC) codes). SIDIAP data is highly representative of the population of Catalonia in terms of geographical, age, and sex distributions [25]. The quality of these data for research use has been previously documented for several diseases [26–28] such as vascular diseases [29], dementia [16], or Alzheimer’s disease [17].

### Definition of AD cases

We identified AD cases using a previously validated algorithm that yielded a positive predictive value of 72.3% (95%CI 70.7–73.9) and a sensitivity of 83.3 (95%CI 81.8–84.6) [17]. This algorithm defined AD cases as (i) patients with a code of AD diagnosis (ICD-10: F00, G30), excluding those who had their AD diagnosis code deregistered or changed for another dementia subtype, or (ii) patients who had a prescription or billing of anti-dementia drugs (ATC codes: N06DA, N06DX01) at any time, excluding those with a history of cerebrovascular disease (ICD-10: I60-I69, G45, G46) up to 2 years before the AD diagnosis, a code for a specific subtype of dementia such as Lewy body dementia and vascular or frontotemporal dementia (ICD-10: F01, F02), or a code for diagnosis (ICD-10: G20–G22) or treatment (ATC: N04) of Parkinson.

### Prevalence and incidence estimates

The estimation of AD prevalence included persons aged 65 years or over, who were alive and registered in SIDIAP on December 31, 2016—persons who died or transferred to another health provider before December 31, 2016, were excluded. Prevalent cases were defined as patients with AD who fulfilled the algorithm conditions on December 31, 2016.

The estimation of the annual incidence rate included persons aged 65 years or over who were alive on January 1, 2016—persons who died, transferred to another health provider, or fulfilled the algorithm conditions for AD before January 1, 2016, were excluded. Incident rate was estimated considering incident cases in the numerator and population at risk in the denominator. Incident cases were defined as patients who fulfilled the algorithm conditions for AD throughout 2016. The denominator was defined as the time contributed by each person registered in SIDIAP throughout 2016, i.e., during the follow-up.

Finally, we described the clinical characteristics of the study population using diagnostic records from SIDIAP database (details of the ICD-10 in Additional file 1); in particular, we provided prevalences of the main risk factors for AD: cardiovascular diseases [30], cerebrovascular diseases [30], anemia [31], cardiovascular risk factors [32], and depression [33].

### Statistical analyses

We used absolute frequencies (percentages) to describe categorical variables and means (standard deviation [SD]) for continuous variables. We calculated prevalence and incidence estimates with a confidence interval (95%CI) based on binomial and Poisson distributions, respectively. Results were stratified by age groups (5-year intervals) and sex. The likelihood ratio (LR) test was used to examine the age-sex interaction. We standardized the estimates using the direct method, and the 2013 Revision of the European Standard Population, which provided the following weights for age groups: 0.28, 0.26, 0.21, 0.13, 0.08, and 0.05 for 65–69, 70–74, 75–79, 80–84, 85–89, and ≥ 90 years, respectively, and a weight of 0.5 for sex groups. Results were replicated in different age populations, including persons aged ≥ 70, ≥ 75, ≥ 80, or ≥ 85 years. All analyses were performed using the R software v3.5.2 [34].

## Results

### Prevalence

To estimate the AD prevalence, we obtained data from 1,048,956 persons registered in SIDIAP on December 31, 2016. The mean age of the study population was 75.9 years (SD = 7.9); they mainly lived in urban areas and were mostly women (Table 1). We detected 39,448 prevalent cases of AD, 28,242 of which were identified by their AD diagnosis and 11,206 by their code for anti-dementia treatment. The crude prevalence was 3.8% (95%CI 3.7–3.8); the age-standardized prevalence, 3.3% (95%CI 3.1–3.5); the sex-standardized prevalence, 3.6% (95%CI 3.5–3.7); and the age- and sex-standardized prevalence, 3.1% (95%CI 2.7–3.6). Prevalence increased with age and was higher in women (Table 2). The increasing trend of prevalence with age was significantly higher in women than in men (LR = 205.10; *p* value < 0.001) (Table 2). Prevalence estimates for different age populations are shown in Additional file 2.

**Table 1:** Characteristics of the study population to estimate the prevalence of Alzheimer’s disease (AD)

Characteristics	Study population (*N* = 1,048,956)	Identified patients with AD (*N* = 39,448)	Other patients (*N* = 1,009,508)
Age, *x̅*_(sd)_	75.9 (7.9)	83.1 (6.6)	75.6 (7.8)
Women, *n* (%)	599,942 (57.2)	28,360 (71.9)	571,582 (56.6)
Rurality, *n* (%)	192,764 (19.1)	8021 (20.3)	200,785 (19.1)
Anemia, *n* (%)	160,524 (15.3)	9852 (25.0)	150,672 (14.9)
Cerebrovascular disease, *n* (%)	107,475 (10.3)	6659 (16.9)	100,816 (10.0)
Coronary heart disease, *n* (%)	100,871 (9.6)	3995 (10.1)	96,876 (9.6)
Diabetes mellitus, *n* (%)	258,214 (24.6)	10,732 (27.2)	247,482 (24.5)
Hyperlipidemia, *n* (%)	567,751 (54.1)	22,144 (56.1)	545,607 (54.1)
Hypertension, *n* (%)	673,663 (64.2)	27,490 (69.7)	646,173 (64.0)
Depression, *n* (%)	175,765 (16.8)	11,222 (28.5)	164,543 (16.3)

**Table 2:** Prevalence of Alzheimer’s disease in Catalonia in 2016 by age groups and sex

	Age group (years)	Cases	Population	Crude prevalence (95%CI)	Standardized prevalence (95%CI)
All population	65–69	1396	282,815	0.5 (0.5–0.5)	0.5 (0.4-0.5)^a^
	70–74	3131	242,888	1.3 (1.2–1.3)	1.3 (1.2–1.4)^a^
	75–79	5885	179,329	3.3 (3.2–3.4)	3.2 (3.0–3.4)^a^
	80–84	11,402	171,494	6.6 (6.5–6.8)	6.3 (6.1–6.6)^a^
	85–89	11,381	110,530	10.3 (10.1–10.5)	9.6 (9.3–10.0)^a^
	≥ 90	6253	61,900	10.1 (9.9–10.3)	9.1 (8.6–9.6)^a^
	**Total**	**39,448**	**1,048,956**	**3.8 (3.7–3.8)**	**3.1 (2.7–3.6)**^**b**^
Women	65–69	850	149,811	0.6 (0.5–0.6)	–
	70–74	1979	131,252	1.5 (1.4–1.6)	–
	75–79	3864	100,054	3.9 (3.7–4.0)	–
	80–84	8093	102,434	7.9 (7.7–8.1)	–
	85–89	8496	71,533	11.9 (11.6–12.1)	–
	≥ 90	5078	44,858	11.3 (11.0–11.6)	–
	**Total**	**28,360**	**599,942**	**4.7 (4.7–4.8)**	**3.8 (3.5–4.2)**^**c**^
Men	65–69	546	133,004	0.4 (0.4–0.4)	–
	70–74	1152	111,636	1.0 (1.0–1.1)	–
	75–79	2021	79,275	2.5 (2.4–2.7)	–
	80–84	3309	69,060	4.8 (4.6–5.0)	–
	85–89	2885	38,997	7.4 (7.1–7.7)	–
	≥ 90	1175	17,042	6.9 (6.5–7.3)	–
	**Total**	**11,088**	**449,014**	**2.5 (2.4–2.5)**	**2.4 (2.1–2.8)**^**c**^

^a^Sex-standardized

^b^Age-sex-standardized

^c^Age-standardized

### Incidence

To estimate the incidence rate, we observed 1,059,422 persons registered in SIDIAP during 2016 (1,032,699 person-years) and identified 4844 incident cases. The crude incidence rate per 1000 person-years was 4.7 (95%CI 4.6–4.8); the age-standardized incidence, 4.3 (95%C,: 4.0–4.6); the sex-standardized incidence, 4.6 (95%CI 4.4–4.7); and the sex- and age-standardized incidence, 4.2 (95%CI 3.8–4.6). The incidence rate was higher in women and increased with age (Table 3), but dropped in the oldest persons (> 90 years). The age-sex interaction was significant, and women showed a higher increment of the incidence rate with age (LR = 53.24; *p* value < 0.001) (Table 3). Incidence rates in persons aged ≥ 70, ≥ 75, ≥ 80, and ≥ 85 years old are provided in Additional file 3.

**Table 3:** Incidence rate (per 1000 person-years) of Alzheimer’s disease in Catalonia in 2016 by age and sex

	Age group (years)	Cases	Person-years	Crude incidence (95%CI)	Standardized incidence (95%CI)
All population	65–69	199	283,590	0.7 (0.6–0.8)	0.7 (0.6–0.9)^a^
	70–74	547	242,267	2.3 (2.1–2.5)	2.2 (2.0–2.5)^a^
	75–79	915	176,418	5.2 (4.9–5.5)	5.1 (4.6–5.6)^a^
	80–84	1555	164,679	9.4 (9.0–9.9)	9.1 (8.5–9.8)^a^
	85–89	1200	104,223	11.5 (10.9–12.2)	11.1 (10.2–12.0)^a^
	≥ 90	428	61,522	7.0 (6.3–7.7)	6.8 (5.9–7.9)^a^
	**Total**	**4844**	**1,032,699**	**4.7 (4.6–4.8)**	**4.2 (3.8–4.6)**^**b**^
Women	65–69	118	149,749	0.8 (0.7–0.9)	–
	70–74	355	130,254	2.7 (2.5–3.0)	–
	75–79	580	97,468	6.0 (5.5–6.5)	–
	80–84	1064	96,633	11.0 (10.4–11.7)	–
	85–89	844	65,875	12.8 (12.0–13.7)	–
	≥ 90	311	43,700	7.1 (6.4–8.0)	–
	**Total**	**3272**	**583,680**	**5.6 (5.4–5.8)**	**4.9 (4.5–5.3)**^**c**^
Men	65–69	81	133,841	0.6 (0.5–0.8)	–
	70–74	192	112,012	1.7 (1.5–2.0)	–
	75–79	335	78,950	4.3 (3.8–4.7)	–
	80–84	491	68,046	7.2 (6.6–7.9)	–
	85–89	356	38,348	9.3 (8.3–10.3)	–
	≥ 90	117	17,822	6.6 (5.4–7.9)	–
	**Total**	**1572**	**449,019**	**3.5 (3.3–3.7)**	**3.5 (3.1–3.9)**^**c**^

^a^Sex-standardized

^b^Age-sex-standardized

^c^Age-standardized

## Discussion

This is the first comprehensive epidemiological study on AD to update prevalence and incidence estimates based on real-world data from primary care. In this respect, we contribute to fulfill a concerning gap in the literature and aim to delve into the epidemiology of AD using real-world data from primary care. Our findings are strengthened by the previous validation of the algorithm used to identify AD cases [17].

Real-word data are increasingly used in overall dementia research [16, 35, 36], but not in the field of AD, where it is surprisingly incipient. To our knowledge, the literature about AD epidemiology based on EHR is limited and restricted to the British population. We only found two studies that used primary care databases to estimate the incidence rate of AD [11, 37], and one study based on EHR from hospitals, which estimated AD prevalence in 5% and incidence rate in 3.7 per 1000 person-years in people aged at least 75 years [38]. Our estimates were higher than the ones reported by the abovementioned study based on EHR from hospitals, since we obtained an AD prevalence of 6.7% and an incidence of 8.1 per 1000 person-years among people aged at least 75 years old. Estimates from primary care databases are expected to be higher than those from secondary care, because there is a time frame in which the diagnosis might only be in the primary care records—if the patient has not yet been admitted to hospital. Surprisingly, AD incidence estimates from previous studies based on primary care databases were more similar to the study based on hospital data than to our estimates of 4.2 per 1000 person-years (95%CI 3.8–4.6). The AD incidence rate was estimated in 1.5 per 1000 person-years using data from The Health Improvement Network database (THIN) [37] and in 1.59 per 1000 person-years using the General Practice Research Database (GPRD) [11], two databases that contain EHR from primary care services in the UK. Our estimate of AD incidence rate (4.2 per 1000 person-years (95%CI 3.8–4.6)) was higher than those in previous studies based on EHR from primary care [11, 37, 38].

Unfortunately, we could not compare our prevalence of AD with similar literature because, to the best of our knowledge, no previous studies based on EHR from primary care have reported such estimates. Other studies were based on EHR, but did not assess AD, only all-cause dementia. For example, Jaakkimainen et al. [39] estimated the prevalence of overall dementia in 72.0 per 1000 persons using EHR recorded by family physicians from Canada. Perera et al. [35] reported prevalences of overall dementia in 2012 of 0.4, 1, 2, 5, 8, 10, and 11 for the following age groups: 65 to 70, 70 to 75, 75 to 80, 80 to 85, 85 to 90, 90 to 95, and ≥ 95 years, respectively. Since AD corresponds to about 50–60% of all dementia cases, we expected to find lower prevalences than the ones observed by Jaakkimainen et al. [39] or by Perera et al. [35], but we obtained estimates similar or slightly lower than the abovementioned prevalences of overall dementia. This could suggest that SIDIAP might capture a higher number of AD and dementia cases than databases from other healthcare systems [35].

The literature on AD epidemiology from face-to-face studies is much more comprehensive than that from EHR studies. Our incidence figures were concordant with the Delphi consensus study that reported a dementia incidence rate of 8.8 per 1000 person-years in Central Europe [1], about 60% of which corresponded to AD [9]. However, our incidence estimates were lower than the observed in two meta-analyses that provided estimates of 15.8 per 1000 person-years worldwide [40] and 11.1 per 1000 person-years in Europe [41]. Notably, both meta-analyses reported significantly high heterogeneity and thus should be interpreted cautiously. In Spain, previous face-to-face studies estimated AD incidence rates of 7.4 per 1000 person-years (95%CI 6.0–8.8) [10] and 10.8 per 1000 person-years (95%CI 7.8–13.7) in people aged at least 65 and 75 years, respectively [42], while our crude estimates were 4.7 (95%CI 4.6–4.8) and 8.09 (95%CI 7.84–8.34) per 1000 person-years in persons aged at least 65 and 75 years, respectively. Our prevalence estimates were slightly lower than the ones reported by face-to-face studies. Age-standardized AD prevalence was estimated in 4.2% worldwide [40], and in 4.4% or 5.1% in Europe [41, 43], while our estimate was 3.3% (95%CI 3.1–3.5). In Spain, age- and sex-standardized prevalence was estimated in 4.0% in people aged ≥ 65 years and in 5.6% in people aged ≥ 75 in face-to-face studies [6, 7]; these findings were slightly lower and similar to our estimates of 3.1% (95%CI 2.7–3.6) in the population aged ≥ 65 years and 5.8% (5.3–6.4) in the population aged ≥ 75 years. Our crude prevalence estimate (3.8% (95%CI 3.7–3.8)) fell within the low range of prevalence estimates from previous studies in the Spanish population, which was from 2.9 to 6.9% [44–47].

Our estimates of prevalence and incidence of AD were similar or slightly lower than face-to-face studies, which could indicate a decreasing trend in AD incidence and prevalence. Previous face-to-face studies were conducted in Spain during the 1990s, and thenceforth, high-income countries have presented a decreasing trend in dementia [3] or AD [48] incidence. However, other countries only observed a decreasing trend in vascular dementia but not in AD [49]; the incidence of AD might actually be increasing, and the decline observed in the incidence of dementia in general could be related to a decline in vascular dementia. Further research to clarify the trend of AD and of vascular dementia would be of interest. Our lower estimates could also be explained by methodological differences, such as population characteristics, different case definitions, or under-registration of AD. In our study, under-registration of AD cases might have occurred for different reasons. First, under-registration is expected to be higher in studies based on EHR than in face-to-face studies, especially in the early stages of AD. In face-to-face studies, patients affected by subclinical symptoms or early stages of AD might likely be identified as AD cases because they are evaluated by specialists during the study. But in studies based on EHR, it is possible that many of these patients have not been cognitively assessed by the end of the study period, and therefore, they are not identified as AD cases. Second, the analysis of free text has been reported to enhance the identification of dementia cases [15]. But we used an algorithm that identified AD cases exclusively based on codes. Therefore, those AD diagnoses recorded in the clinical history using the free text instead of the ICD-10 codes enhanced under-registration in our study. Third, patients diagnosed by a specialist from private healthcare might not have the AD diagnosis recorded in their clinical history, unless they (or their relatives) inform the general practitioner about their condition. Despite the universal coverage of the Catalan public health system, about 25% of persons aged at least 60 years have contracted a private health insurance. However, not all of them might have contributed to under-registration in our study. Most of the private healthcare users also consult public healthcare professionals, especially among the elderly. The public health system provides free access or low copayment for pharmacological treatment prescribed by general practitioners to all Catalan pensioners. In our study, we identified AD cases combining data recorded in the primary care settings with data from the pharmacy-invoicing database. Therefore, we could identify as AD cases those patients attended by a private specialist who asked their general practitioner for prescription of anti-dementia drugs to benefit from economic discounts or for a prescription renewal.

In SIDIAP, under-registration of AD was concentrated in the oldest group: we observed increasing trends of AD prevalence and incidence with age except for this group (≥ 90 years), in line with previous studies [50]. Some general practitioners might be reluctant to use dementia codes among the oldest persons because they may consider the diagnosis and treatment as ineffectual in this population or may interpret their memory difficulties as part of the normal aging process rather than as a disability [16]. Besides, people aged at least 90 years might have higher levels of comorbidity that could affect the quality of AD diagnosis and register. For example, those persons who have impaired mobility due to chronic complex diseases might not be able to visit the specialist, resulting in a diagnosis of unspecified dementia instead of AD; this is in line with previous findings that reported the highest prevalence and incidence rate estimates of overall dementia among the oldest elderly in Catalonia [16]. Patients institutionalized in private geriatrics or residences might lose contact with their general practitioner, increasing under-registration of EHR. Moreover, patients with vascular comorbidities who had a diagnosis of AD and were diagnosed with another dementia subtype (such as mixed dementia or vascular dementia) were excluded from our study, because the algorithm we used only defined dementia cases as AD if this was the last diagnosis. Finally, certain aspects other than under-registration could partially explain our lower estimates in the oldest elderly: some level of survival bias that could not be rejected and an attenuation of the relationship between AD diagnosis and clinical expression of dementia in the oldest old, which has been pointed out in recent studies [49].

Our estimates were clearly higher than those in previous studies based on EHR, suggesting that under-diagnosis in primary care settings probably varies strongly depending on the clinical practice. In the Catalan primary care, underestimation appeared to be less frequent than in other primary care settings, which reported that only half of the expected number of patients with dementia were recognized by the general practitioner [14]. This could be partly explained because the accuracy of AD diagnosis routinely collected in primary care might improve in active dementia diagnosis centers; in our study settings, about 25% of patients without dementia and 75% of patients with dementia aged 65 years or over have taken a cognitive test [16]. Additionally, about 90% of the AD prevalent cases recorded in primary care had been evaluated by a specialist, which suggests that general practitioners are coordinated with the secondary care settings [17]. This coordination is promoted by initiatives like an asynchronous telemedicine program that establishes protocols for the screening and diagnosis of dementia, thereby enabling primary care and specialized care professionals to reach shared diagnoses of dementia [51]. Finally, under-registration in our study was probably moderate because we might have captured a high number of AD cases among treated patients. In Catalonia, about 68% of dementia patients receive anti-dementia drugs [52], and this percentage may be higher than in other countries: in France, it is about 38% [53], and in Germany, it is about 52% [54]. Furthermore, our definition of AD case might have contributed to increase the number of AD cases identified by anti-dementia treatment. We included patients treated with anti-dementia drugs at any time—that is, patients who were treated before the study period were also included—whereas other studies accounted for currently treated patients—patients who were medicated in the past were excluded.

We acknowledge some limitations. First, our results could be underestimated because we did not use free text recorded in the clinical history, an accredited method to identify persons with AD [15]. However, we used a validated algorithm to identify AD cases [17] that combined diagnosis with prescription or dispensing codes, involving not only data from general practitioners but also from a pharmacy*-*invoicing database. In Catalonia, the prescription of anti-dementia drugs is requested by the general practitioner but requires approval by certain other specialists (geriatricians, psychiatrists, or neurologists). The use of two different data sources minimized the possible under-recording of dementia diagnoses in primary care. Second, the use of certain additional clinical parameters to diagnose AD could have increased the number of identified AD cases. However, we prioritized the use of a simple algorithm that was validated and had a good positive predictive value (72.3%, 95%CI 70.7–73.9), and sensitivity (83.3, 95%CI 81.8–84.6) [17]. Third, we did not account for educational level, a risk factor for AD, because such information was not available in SIDIAP. Fourth, AD is one of the many dementia disorders, and epidemiological trends might vary between dementia subtypes, for example, decreasing trends of overall dementia have been reported due to the reduction of vascular dementia but not of AD [49]. Thus, further details on the epidemiology of other dementia subtypes are crucial to plan effective preventive measures against dementia.

The main strength of our study is our large sample size (including more than 1 million persons aged at least 65 years old, and 40,000 dementia cases), which enhanced external validity; SIDIAP contains high-quality data from about 80% of the Catalan population and good representativeness in terms of geographical, age, and sex distributions [25].

## Conclusions

The prevalence and incidence rates of AD based on EHR from primary care were higher than other estimates based on primary care databases from other European countries and slightly lower than the most recent face-to-face studies. Despite some limitations, real-world data routinely collected in primary care settings could be applied to epidemiological studies to complement face-to-face studies and could provide regular updates of prevalence and incidence estimates of AD at a reasonable cost. Better monitoring of the AD trends might help adjust the national dementia plans and the allocation of resources for families and caregivers to the current number of cases occurring in the community. Additionally, our results contribute to raise awareness about the importance of the accuracy of EHR and might encourage general practitioners, nurses, and specialists to keep improving the quality of the data they routinely collect in the electronic health histories. It is time to use real-world data from the primary care settings to update AD epidemiology complementing face-to-face studies, which might contribute to improve dementia policies and resource management for patients and their families.

## Supplementary information

**Additional file 1. **Diagnostic and pharmacological codes. Description of data: Definition of comorbidities based on the *International Classification of Diseases, 10th revision* (ICD-10).

**Additional file 2.** Prevalence of AD. Prevalence of Alzheimer’s disease in the Catalan population in 2016 in different age-specific populations (≥ 70, ≥ 75, ≥ 80 and ≥ 85 years old), by age groups and sex.

**Additional file 3.** Incidence of AD. Description of data: incidence rate of Alzheimer’s disease in the Catalan population in 2016 in different age-specific populations (≥ 70, ≥ 75, ≥ 80 and ≥ 85 years old), by age groups and sex.

## Data Availability

The datasets generated during and/or analyzed during the current study are not publicly available due to legal reasons related to data privacy protection.
