# Supplementary material for: Is it time to use real-world data from primary care in Alzheimer’s disease?
Source: Alzheimers Res Ther. 2020 May 18;12:60. doi: 10.1186/s13195-020-00625-2 (PMC7236302; doi:10.1186/s13195-020-00625-2)
Supplement: Supplementary file 1 — Additional file 1. Diagnostic and pharmacological codes. Description of data: Definition of comorbidities based on the International Classification of Diseases, 10th revision (ICD-10). [file 13195_2020_625_MOESM1_ESM.docx]

# Additional file 1

Definition of comorbidities based on the *International Classification of Diseases, 10^th^ revision* (ICD-10).

Anemia: D46*, D50*-D53*, D55*, D57*-D59*, D61*-D64*

Cerebrovascular disease: G10, G11*-G13*, G30*-G32*, G35-G37*, G45, G90*-G99*, I60*-I63*, I64, I67*, I69*

Coronary heart disease: I20*-I22*, I24*, I25*, Z95.1

Diabetes mellitus: E10*-E14*

Hyperlipidemia: E78*

Hypertension: I10, I11*-I13*, I15*

Depression: F30*-F34*, F38*, F39

*All subtypes included.
