# Supplementary material for: Is it time to use real-world data from primary care in Alzheimer’s disease?
Source: Alzheimers Res Ther. 2020 May 18;12:60. doi: 10.1186/s13195-020-00625-2 (PMC7236302; doi:10.1186/s13195-020-00625-2)
Supplement: Supplementary file 2 — Additional file 2. Prevalence of AD. Prevalence of Alzheimer’s disease in the Catalan population in 2016 in different age-specific populations (≥ 70, ≥ 75, ≥ 80 and ≥ 85 years old), by age groups and sex. [file 13195_2020_625_MOESM2_ESM.docx]

# Additional file 2

Prevalence of Alzheimer’s disease in the Catalan population in 2016 in different age-specific populations (≥ 70, ≥ 75, ≥ 80 and ≥ 85 years old), by age groups and sex.

| **Age populations** | **Sex** | **Cases** | **Population** | **Crude Prevalence (95%CI)** | **Standardized Prevalence (95%CI)** |
| --- | --- | --- | --- | --- | --- |
| ≥70 | Total | 38,052 | 766,141 | 4.97 (4.91-5.02) | 4.19 (3.72-4.72)^a^ |
|  | Women | 27,510 | 450,131 | 6.11 (6.04-6.18) | 5.13 (4.80-5.49)^b^ |
|  | Men | 10,542 | 316,010 | 3.34 (3.27-3.40) | 3.24 (2.90-3.62)^b^ |
| ≥75 | Total | 34,921 | 523,253 | 6.67 (6.61-6.74) | 5.81 (5.29-6.39)^a^ |
|  | Women | 25,531 | 318,879 | 8.01 (7.91-8.10) | 7.15 (6.78-7.53)^b^ |
|  | Men | 9,390 | 204,374 | 4.59 (4.50-4.69) | 4.46 (4.08-4.88)^b^ |
| ≥80 | Total | 29,036 | 343,924 | 8.44 (8.35-8.54) | 7.89 (7.32-8.50)^a^ |
|  | Women | 21,667 | 218,825 | 9.90 (9.78-10.03) | 9.78 (9.39-10.18)^b^ |
|  | Men | 7,369 | 125,099 | 5.89 (5.76-6.02) | 5.99 (5.58-6.45)^b^ |
| ≥85 | Total | 17,634 | 172,430 | 10.23 (10.08-10.37) | 9.43 (8.85-10.04)^a^ |
|  | Women | 13,574 | 116,391 | 11.66 (11.48-11.85) | 11.65 (11.28-12.04)^b^ |
|  | Men | 4,060 | 56,039 | 7.25 (7.03-7.46) | 7.20 (6.76-7.66)^b^ |

^a^Age-sex-standardized; ^b^age-standardized.
