# Supplementary material for: Is it time to use real-world data from primary care in Alzheimer’s disease?
Source: Alzheimers Res Ther. 2020 May 18;12:60. doi: 10.1186/s13195-020-00625-2 (PMC7236302; doi:10.1186/s13195-020-00625-2)
Supplement: Supplementary file 3 — Additional file 3. Incidence of AD. Description of data: incidence rate of Alzheimer’s disease in the Catalan population in 2016 in different age-specific populations (≥ 70, ≥ 75, ≥ 80 and ≥ 85 years old), by age groups and sex. [file 13195_2020_625_MOESM3_ESM.docx]

# Additional file 3

Incidence rate (per 1000 persons-year) of Alzheimer’s disease in the Catalan population in 2016 in different age-specific populations (≥ 70, ≥ 75, ≥ 80 and ≥ 85 years old), by age groups and sex.

| **Age populations** | **Sex** | **Cases** | **Person/years** | **Crude Incidence (95%CI)** | **Standardized Incidence (95%CI)** |
| --- | --- | --- | --- | --- | --- |
| ≥70 | Total | 4,645 | 749,109 | 6.20 (6.02-6.38) | 5.55 (5.05-6.09)^a^ |
|  | Women | 3,154 | 433,931 | 7.27 (7.02-7.53) | 6.52 (6.02-7.05)^b^ |
|  | Men | 1,491 | 315,178 | 4.73 (4.49-4.98) | 4.58 (4.07-5.13)^b^ |
| ≥75 | Total | 4,098 | 506,842 | 8.09 (7.84-8.34) | 7.40 (6.76-8.08)^a^ |
|  | Women | 2,799 | 303,676 | 9.22 (8.88-9.56) | 8.63 (8.01-9.29)^b^ |
|  | Men | 1,299 | 203,166 | 6.39 (6.05-6.75) | 6.17 (5.51-6.88)^b^ |
| ≥80 | Total | 3,183 | 330,424 | 9.63 (9.30-9.97) | 9.24 (8.46-10.08)^a^ |
|  | Women | 2,219 | 206,208 | 10.76 (10.32-11.22) | 10.77 (10.04-11.55)^b^ |
|  | Men | 964 | 124,216 | 7.76 (7.28-8.27) | 7.71 (6.88-8.61)^b^ |
| ≥85 | Total | 1,628 | 165,745 | 9.82 (9.35-10.31) | 9.37 (8.45-10.37)^a^ |
|  | Women | 1,155 | 109,575 | 10.54 (9.94-11.17) | 10.53 (9.72-11.41) ^b^ |
|  | Men | 473 | 56,170 | 8.42 (7.68-9.22) | 8.20 (7.18-9.33)^b^ |

^a^Age-sex-standardized; ^b^age-standardized.
